# Combustion analysis of CI engine fuelled with calophyllum inophyllum methyl ester biodiesel and CeO_2_ nanoparticle additives

**DOI:** 10.1038/s41598-025-29087-y

**Published:** 2025-12-29

**Authors:** Vivek Pandey, Irfan Anjum Badruddin, Mohammad Zuber, Sarfaraz Kamangar

**Affiliations:** 1 Mechanical Engineering Department, Dr. C. V. Raman University, Bilaspur, 495113 Chhattisgarh India; 2 Mechanical Engineering Department, College of Engineering, King Khalid University, Abha, Asir, 61413 Saudi Arabia; 3 Department of Aeronautical & Automobile Engineering, Manipal Institute of Technology, Manipal Academy of Higher Education, Manipal, 576104 India

**Keywords:** Biodiesel, Calophyllum inophyllum, Cerium oxide nanoparticles, Combustion stability, Cyclic variability, Ignition delay, Cetane number, Mechanical engineering, Biodiesel

## Abstract

This study investigates the effect of cerium oxide (CeO_2_​) nanoparticle (NP) size on the combustion characteristics of a single-cylinder compression ignition (CI) or diesel engine fueled with a base blend (BD) composed of 20% Calophyllum Inophyllum Methyl Ester biodiesel (CIMEBD), and 80% diesel (by volume). The CIMEBD used in this study was synthesized via a two-stage transesterification process from crude Calophyllum Inophyllum oil. The challenges related to biodiesel properties such as viscosity and oxidation stability can limit the use of biodiesel blends to less than 20% biodiesel. As a result, 20%-biodiesel, 80%-diesel is a common and widely approved blend for use in modern diesel engines without modification, as per regulatory standards, such as the ASTM D7467 standard. NPs can be used with biodiesels in order to counteract their inherent higher viscosity, and thereby allowing the application of higher biodiesel percentages. CeO_2_​ NP of four different average sizes (20, 40, 60, and 80 nm) were dispersed in the base biodiesel blend, BD at a fixed concentration of 90 ppm. The engine was operated at a constant speed of 1500 RPM under various loads. Key combustion parameters, including in-cylinder pressure, heat release rate (HRR), ignition delay (ID), cetane number (CN), and the coefficient of variation of indicated mean effective pressure (COVIMEP) were analyzed. Results show that the addition of NPs improves combustion stability and performance. The fuel blend with 40 nm NPs (BD40) exhibited the most favorable characteristics, demonstrating the lowest cyclic variability (COVIMEP​ of 1.9% at 30% load, compared to 3.1% for diesel) and the shortest ID, in crank angle degrees (CAD), that is, 3.14 CAD at 30% load for base biodiesel, vs. 4.5 CAD for diesel. This was attributed to the BD40 blend having the highest measured CN (55.4). A strong inverse correlation was established between CN, ID, and COVIMEP​. The findings indicate that an optimal NP size of 40 nm exists to maximize the catalytic benefits for biodiesel combustion, with agglomeration effects potentially diminishing the performance of larger NPs, thus establishing a clear, size-dependent relationship for combustion stability.

## Introduction

Internal combustion engines (ICE) are the prime movers of the present age and will continue to remain so in the near future^1,2^. Among ICE, diesel engines are the predominant transport vectors, carrying more than 98% of cargo. These engines have desirable characteristics including the absence of throttling losses characteristic of spark-ignition engines, high thermal efficiency, suitability for turbocharging, operation at high compression ratios, and minimal knock issues^3,4^. This combination of robustness and efficiency has cemented their role as the backbone of global logistics and heavy-duty transportation.

However, the operational paradigm of these engines is facing unprecedented challenges. More than 96% of ICEs run on fossil-derived fuels. It is known that these fuels are non-renewable^5,6^ and are expected to be depleted soon in the near future^7^. Moreover, ICEs and diesel engines in particular are responsible for predominantly emitting harmful emissions^8^, and emissions are being scrutinised under increasingly stringent global regulations. With the predicted increase in global fuel consumption [9], coupled with the “net zero emissions by 2050” initiative, there is a focus now on the development of renewable substitutes for fossil fuels. This imperative has catalysed extensive research into a spectrum of alternatives. In this context, various alternative gaseous^10–12^ and liquid fuels^13–16^ are being investigated.

Among these alternatives, biodiesels are seen as viable substitutes for diesel engine fuel as they address some of the issues mentioned and are being investigated intensively^17–22^. Biodiesels offer a pathway to carbon-neutral operation and can often be used in existing engine hardware with minimal modification. A particularly promising source for next-generation biodiesel is the Calophyllum Inophyllum plants, whose seeds are used to produce Calophyllum Inophyllum Methyl Ester Biodiesel (CIMEBD), and are found primarily in the Asian subcontinent^23^. This source holds a distinct strategic advantage, as they are high oil yielding, non-edible plant species and can survive in harsh terrain^24^. This circumvents the critical “food versus fuel” debate that has shadowed first-generation biofuels derived from food crops, as it does not compete for arable land needed for food production.

Biodiesel from Calophyllum Inophyllum comes from the oil obtained from its plants’ seeds, the oil being converted to methyl esters. For the present investigation, CIMEBD was produced via a two-stage transesterification process and its key physicochemical properties were measured according to ASTM standards. Table 1 presents these measured properties and compares them against the standard specifications for conventional diesel fuel (ASTM D975) and biodiesel blend stock (ASTM D6751) to ensure compliance and provide a clear basis for comparison.

**Table 1:** Physicochemical properties of test fuels.

Property	Test method	Diesel (from supplier)	CIMEBD (measured)	ASTM D975 (No. 2-D)
Kinematic Viscosity @ 40 °C (mm²/s)	ASTM D445	2.8	4.9	1.9–4.1
Density @ 15 °C (kg/m³)	ASTM D1298	845	875	850
Lower Calorific Value (MJ/kg)	ASTM D240	44.8	39.1	42.7
Cetane Number	ASTM D613	49.1	51.6	> 40
Flash Point (°C)	ASTM D93	72	158	> 52
Oxidation Stability @ 110 °C (h)	EN 15,751	> 20	4.1	--

Note: The values for Diesel and CIMEBD are the measured properties of the specific fuels used in this experimental work.

The data in Table 1 frames the potential and challenges of CIMEBD. Its higher cetane number (CN) at 51.6 suggests favourable ignition quality, while its significantly higher flash point (158 °C) indicates superior handling safety. Conversely, its lower calorific value (LCV) at 39.1 MJ/kg signals an inherent energy deficit compared to diesel, which is expected to influence fuel consumption and power output. Furthermore, its kinematic viscosity (4.9 mm^2^/s) is higher than that of diesel and falls outside the upper limit of the ASTM D975 standard, a common characteristic of biodiesels that can impact fuel atomization and spray characteristics. Among the general class of biodiesels, CIMEBD has relatively superior oxidation stability and forms a more stable fuel suspension compared to other biodiesels. In addition, it has a lower viscosity compared to biodiesels from other raw oils that would account for a comparatively finer atomization and reduced injector clogging^25–27^. Furthermore, compared to other biodiesels, CIMEBD can provide enhanced combustion by reduction in ignition delay (ID), higher molecular oxygen content, and better brake thermal efficiency (BTE)^28–31^.

In order to further enhance the viability of biodiesels, advanced formulation strategies are required. This has led to the exploration of nanotechnology as a means to augment fuel properties and optimize the combustion process.

### A critical review of fuel additives for enhanced engine characteristics

The inherent limitations of biodiesels, such as higher viscosity and lower energy content, can be mitigated through the use of fuel additives. Among the most promising class of additives are nanoparticles (NPs), which are in the 1–100 nm size range, and when dispersed in a base fuel, create engineered fluids known as nano-fuels with tailored properties.

#### NPs as catalytic fuel additives: mechanisms and effects

The disadvantages of biodiesels can be overcome by blending them with NPs to form colloidal mixtures that are known to improve the fuel properties, engine performance^32^, and combustion characteristics^33,34^. An engineered fuel known as a ‘nano-fuel’ is created by dispersing NPs in a base fuel to form these “colloidal mixtures”^35^.

The efficacy of these NPs stems from two primary mechanisms. First, the fundamental physics of their nanoscale dimensions provides a powerful catalytic advantage. Due to the high surface to volume ratio of NPs, there are multiple reaction sites that account for their catalytic activity^36^. This vastly increased reactive surface area allows a small mass of NPs to exert a significant chemical influence on the combustion process. Second, NPs introduce a unique physical phenomenon that directly enhances fuel preparation within the cylinder. NPs are known for the ‘micro-explosion’ phenomenon which enhances local fuel air mixing, and thereby enhances combustion performance^37,38^. This process involves the rapid, disruptive boiling of volatile components within a fuel droplet around a nanoparticle nucleus, leading to the violent shattering of the primary droplet into a fine mist of smaller secondary droplets. This secondary atomization fundamentally improves the fuel-air mixing process, which is critical for complete and efficient combustion. Experimental work has provided compelling validation of this mechanism^39^. It was observed that while single droplet evaporation of nano-fuels conformed to the classical D^2^ law at lower temperatures, this behaviour was disrupted at higher temperatures. At the higher temperature, evaporation was not in conformation to the D^2^ law; this result being accentuated for higher NP concentration, or lower average NP size. The cause was attributed to NP as nucleation sites that are responsible for “enhanced expansion and micro-explosion intensities”, thereby promoting fuel droplet evaporation. This micro-explosion effect is particularly beneficial for high-viscosity fuels like biodiesel, as it physically compensates for potentially poorer primary atomization at the injector nozzle.

Among the different NPs in use, Cerium oxide (CeO_2_)​ NPs are being applied in engine research due to their unique chemical properties. CeO_2_​ NPs are known to easily transform from the Ce + 3 to Ce + 4 valence states in their respective compounds, which assists in easy storage and supply of oxygen during combustion. Therefore, they are good oxidative catalysts^40^. This ability to act as an “oxygen buffer”—releasing oxygen in fuel-rich zones and absorbing it in fuel-lean zones—makes it a potent oxidative catalyst that promotes more complete oxidation of carbon monoxide (CO) and unburnt hydrocarbons (UHC).

#### Performance and emission outcomes of Nano-fuel formulations in diesel engines

In the context of NP additives for nano-fuel synthesis, and nano-fuel application to diesel engine research, numerous reviews and research articles are available^35,40–44^, that describe the potential of NP additives towards improving the performance of engines running on biodiesel and biodiesel-diesel blends.

NPs are known to improve the poor combustion characteristics of biodiesel due mainly to their larger molecular weight and lower calorific values^40^. The collective body of research indicates that due to many of their favourable properties, NPs also assist in the reduction of engine emissions^40,45^.

However, a closer examination of the literature reveals a complex and sometimes contradictory picture, particularly concerning nitrogen oxide (NOx​) emissions. The formation of NOx​ is highly sensitive to a confluence of competing factors, including peak combustion temperature, oxygen availability, and residence time.

NP addition influences all of these factors, leading to variable outcomes. On one hand, some researchers report significant NOx​ reductions, with some review results pointing to improvements in engine performance and a decrease in all emissions of concern, including NOx​^40,45^. This is supported by experimental work^46^ that observed a remarkable reduction in NOx ranging from 25 to 30% with use of NP additives (Graphene Oxide and Graphene Nanoplatelets) in Karanja and waste cooking oil biodiesels. Conversely, a substantial portion of the literature reports either an increase or no significant change in NOx​. A comprehensive review^35^ observed that results for NOx are variable depending on the operating conditions as well as the fuel type and NP employed. In most studies, however, NOx increments have been reported as per the review. This finding is echoed in experimental work^47^ that investigated lemon-peel and orange-peel biodiesels with MWCNT and CeO_2_​ NPs and found an overall reduction in emissions, except for NOx was observed. The challenge of managing NOx​ with biodiesel is so significant that it has been the subject of dedicated reviews, which present nanoparticle additives as one potential solution alongside engine control strategies like ignition retard and high-rate exhaust gas recirculation (EGR)^44^.

This apparent contradiction in the literature regarding NOx emissions highlights a key methodological challenge in nano-fuel research. The final NOx emission is the result of competing phenomena (e.g., higher peak temperatures vs. altered combustion phasing). Many studies fail to deconvolve these effects, making direct comparisons between different nanoparticles and base fuels difficult and leading to inconsistent conclusions.

This is indicative of a delicate balance at play. Improved combustion efficiency, driven by the catalytic effect of NPs, can lead to higher in-cylinder temperature, a factor that promotes thermal NOx​ formation. Simultaneously, NP-induced changes to the combustion phasing and heat release rate (HRR) can shorten the premixed combustion phase and lower the peak temperature, these factors causing the suppression of NOx​ formation. The final net NOx​ emission is the result of these competing phenomena, which is highly dependent on the specific NP type, concentration, base fuel, and engine operating conditions. In contrast to the variability of NOx​, reductions in CO, UHCs, and smoke opacity are more consistently reported across studies.

Furthermore, modern computational methods are being leveraged to tackle the optimization challenge of engine emissions versus engine performance. AI-driven approaches, for example, have been successfully used to optimize engine performance and emissions in urban commercial vehicles, showcasing a powerful alternative to purely experimental iteration^48,49^.

In a similar vein, artificial intelligence-based models have been developed to predict the energy efficiency and emissions of engines fueled with soybean methyl ester under various compression ratios, demonstrating the power of computational tools in this field^50^.

Other approaches move beyond standard performance metrics to employ more comprehensive analytical frameworks. For example, the addition of oxyhydrogen to palm oil biodiesel has been evaluated not just for performance, but through detailed energy, exergy, and sustainability analyses to provide a more holistic assessment of the fuel’s viability^51^.

Alongside the use of NP additives, another significant area of research involves the formulation of complex, multi-component fuel blends. For instance, studies have evaluated the performance and emission characteristics of engines fueled with blends of plastic-derived oil, hydrogen, and diesel, demonstrating how engine operating parameters like fuel injection strategy are critical to optimizing the combustion of such advanced fuels^52^.

Further research has explored even more complex fuel strategies, such as combining hydrogen fumigation with nano-additives in biodiesel blends under EGR conditions to simultaneously optimize performance and emissions^53^.

Similarly, the effects of adding alcohols to complex biodiesel-diesel-hydrogen fuel mixtures have been investigated through both experimental work and parametric modeling to understand the intricate interactions affecting engine characteristics^54^.

Moreover, the impact of biofuels extends to transient operating conditions, with studies characterizing the specific nature of hazardous particulate emissions during cold-start and warm-up phases, highlighting the influence of both the biofuel and lubricating oil on particle mass and number^55^.

#### Investigations into the combustion characteristics of NP-dosed biofuels

In order to understand the root causes of the observed performance and emission outcomes, it is essential to analyse the underlying combustion phenomena. A number of studies have investigated key combustion parameters such as in-cylinder pressure, HRR, ID, and combustion duration^46,47,56–61^.

A widely reported and consistent finding is that NP addition advances the start of combustion. Multiple independent investigations have shown that nano-fuels lead to a shorter ID. One study^46^ noted a maximum reduction of 1.6 in ID for 60 ppm GNP, 20% biodiesel fraction. Another^56^ found that the ignition advance with MoO_3_​ NPs was 1 CAD, whereas that for CNT NPs was about 1.85. Similar ignition advance was reported using CeO_2_​ NPs^59,60^. This reduction in ID is a direct result of the enhanced catalytic activity of NPs^36^, which lowers the activation energy required for the onset of combustion.

The effect of NPs on the subsequent shape of the combustion event—specifically the peak in-cylinder pressure and peak HRR—is more varied. Many studies report an increase in these parameters, viewing it as a direct indicator of more rapid and complete energy release. An ignition-advance and rise in both HRR and pressure were observed with CeO_2_-NP diesel blends^59^. It was similarly found that in-cylinder pressure and HRR peaks were higher with higher NP concentration^60^. Another study^61^ noted that peak combustion pressures were higher with NPs compared to those without NPs.

However, an alternative narrative suggests that a smoother, less aggressive heat release profile may be more desirable. One investigation^56^ found that while CNT and MoO_3_​ NPs aided in advanced ignition, the peak HRRs were reduced by 10% and 15% for MoO_3_​, and CNT NPs, respectively. The argument is that the earlier start of combustion allows the energy to be released over a slightly longer duration, preventing an overly sharp pressure spike that can increase engine noise and mechanical stress. This perspective is complicated by the fuel’s energy content. It was found that the HRR for Palm stearin biodiesel (PSBD)-NP blends was lower compared to diesel^61^, attributed to the lower calorific value of the biodiesel, a direct link to the fundamental fuel properties shown in Table 1. This highlights a critical ambiguity: a lower peak HRR can be interpreted as either a sign of smoother, more controlled combustion or simply a consequence of lower fuel energy density. The optimal HRR profile is therefore not necessarily the one with the highest peak, but one that is optimally shaped to maximize thermal efficiency while minimizing noise, engine vibration and NOx​ formation.

#### Prior research on CIMEBD and related nano-fuel blends

Research specifically involving CIMEBD has demonstrated some potential. Studies have shown that CIMEBD enhances combustion by reducing the ID thereby causing the pressure peak to be attained earlier and results in better engine torque at a given RPM, compared to some other biodiesels^28^. The reduced BTE from CIMEBD can be countered by use of higher injection pressures^30^. The inherent molecular oxygen in CIMEBD is known to assist the enhancement of combustion^31^.

Despite the established promise of CIMEBD as a second-generation biofuel and the extensive research into CeO_2_​ as a leading NP additive, the intersection of these specific materials remains largely unexplored. A thorough review of the literature reveals only one prominent study that has investigated CIMEBD in a nano-fuel formulation. This study^58^ used Graphene Oxide (GO) NPs and CIMEBD (20%)-Diesel (80%) blends to investigate CI engine characteristics. The authors reported that regulated emissions (NOx, UHC, CO, CO, smoke) showed reduction with 50 ppm GO NPs, and also noted reductions in several unregulated emissions. Critically, some results relating to HRR, combustion delay, and in-cylinder pressure were also presented.

This work serves as a crucial proof of concept, demonstrating that CIMEBD is a viable base fuel for nanofluid applications. However, it also highlights a conspicuous gap in the research landscape. A dedicated investigation combining the promising CIMEBD fuel with the highly effective CeO_2_​ catalyst appears to be absent from the published literature, especially one that focuses in detail on the resulting combustion characteristics.

Since the performance and emission characteristics of the fuel blends used herein have been reported by the authors in a previous study^62^, the present work provides exclusive focus on the in-depth analysis of the combustion characteristics to understand the underlying mechanisms responsible for the previously observed performance and emission trends, with a primary focus on combustion stability.

### Research gap and objectives

The literature confirms the potential of both CIMEBD as a fuel and CeO_2_ NPs as additives. However, there is a distinct lack of research systematically investigating the effect of nanoparticle size on the cyclic combustion variability of a CIMEBD-fueled engine. Cyclic variability, quantified by COVIMEP, is a critical parameter for engine stability, yet it remains largely unexplored in the context of CIMEBD nano-fuels, as highlighted in Table 2, which shows summary of key literature on nano-fuel additives in CI Engines in the context of combustion stability. Cyclic variability, often quantified by the coefficient of variation of indicated mean effective pressure (COVIMEP​), is a critical parameter for engine stability, especially when exploring advanced combustion strategies.

This represents a specific and significant gap at the confluence of two promising research avenues, especially focused on engine combustion characteristics.

Therefore, in this work, the authors have proposed to study a 4-stroke, naturally aspirated and water-cooled diesel engine using CIMEBD and CeO_2_​ NPs and investigate the engine combustion characteristics. The primary goal is to provide the first detailed analysis of this specific nanofuel combination, focusing on the fundamental in-cylinder phenomena that govern engine performance and emissions.

Based on the research gaps, this study hypothesizes that an optimal CeO_2_ nanoparticle size exists that will minimize ignition delay and, consequently, the cyclic combustion variability (COVIMEP) of CIMEBD blends. We further hypothesize that performance will diminish for particles larger than this optimum size due to increased agglomeration, which reduces catalytic efficiency. Therefore, the primary objective of this study is to systematically investigate the effect of CeO_2_ nanoparticle size on the fundamental combustion characteristics, particularly combustion stability, of a CI engine fueled with a CIMEBD-diesel blend. Its novelty lies in providing the first detailed analysis of this specific nanofuel combination, with a focus on elucidating the quantitative relationship between nanoparticle size, cetane number, ignition delay, and cyclic variability (COVIMEP).

The specific objectives of this study are:

- To prepare stable nanofuel blends consisting of a CIMEBD-Diesel base fuel doped with CeO_2_​ NPs.
- To experimentally investigate the effect of these nanofuel blends on the fundamental combustion characteristics of a single-cylinder, constant-speed diesel engine under varying load conditions.
- To conduct a comparative analysis of key combustion parameters—including in-cylinder pressure, HRR, ID, and combustion duration—against baseline operation with conventional diesel and an un-doped CIMEBD-diesel blend.
- To elucidate the specific catalytic and physical effects of the CeO_2_​ NPs on the combustion process of CIMEBD, thereby contributing novel data to address the identified gap in the scientific literature.

**Table 2:** Summary of key literature on nanofuel additives in CI Engines.

Reference	Fuel type	Nanoparticle additive	Combustion stability (COVIMEP​) investigated?	Key focus
Anbarsooz^35^	Diesel/Biodiesel	Various	No	Review of performance and emissions.
Hoang^40^	Diesel/Biodiesel	CeO_2_​	No	Review of CeO2​ effects on performance/emissions.
Chacko and Jeyaseelan^46^	Karanja/WCO Biodiesel	GO, GNP	No	Combustion (ID, HRR) and emissions.
Mei et al.^56^	Diesel	CNT, MoO_3_​	No	Combustion (ID, HRR) and emissions.
Sheriff et al.^47^	Lemon/Orange Peel Biodiesel	MWCNT, CeO2​	No	Combustion (ID, HRR) and emissions.
Gowtham and Prakash^58^	CIMEBD	GO	No	Regulated and unregulated emissions.
Devarajan et al.^61^	Palm Stearin Biodiesel	AgO (5, 10, 20 nm)	No	Effect of NP size on pressure and HRR.
Present study	CIMEBD	CeO_2_; (20, 40, 60, 80 nm)	Yes	Effect of NP size on combustion stability (COVIMEP​).

## Materials and methods

**Fig. 1:**
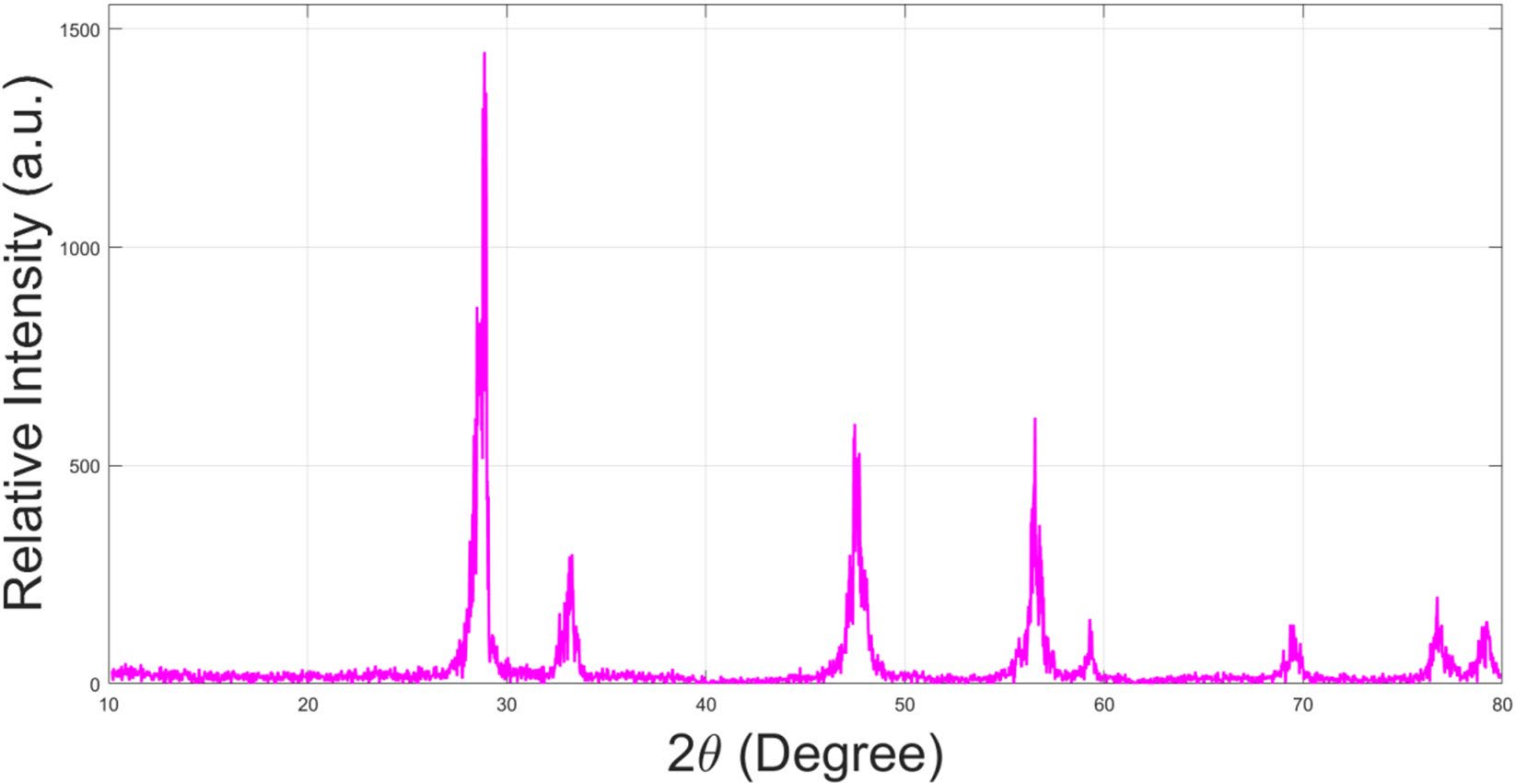
XRD Intensity versus 2θ indicating Ceria NP.

The manufacture and characterization of CeO_2_ NP is shown in our earlier published research article^62^, and briefly described in the next subsection. Figure 1 displays the X-Ray Diffraction (XRD) spectra for Ceria NP. “CuKα radiation (0.15 nm) was used for the characterization. Diffraction peaks for the NPs indicate the presence of Ceria (CeO_2_) as per Standards of the Joint Committee for Powder Diffraction Studies (JCPDS) File No. 34-0394. The average NP size was found from Scherrer’s^63^ equation.”

### Nanoparticle synthesis and characterization

The Cerium Oxide (CeO_2_) nanoparticles were synthesized in-house using a co-precipitation method, with full details provided in our prior work^62^. Briefly, an aqueous solution of cerium nitrate hexahydrate served as the precursor, and ammonium hydroxide was added dropwise as the precipitating agent under constant stirring. The resulting precipitate was washed, dried, and calcined at 500 °C for 4 h to obtain the final CeO_2_ nanopowder. The crystalline structure was confirmed by XRD analysis (Fig. 1), and the average particle size was determined using the Scherrer equation^63^. CIMEBD was synthesized using a two-stage process since the free fatty acid (FFA) content in oils is >4%^64^. The synthesized CIMEBD has properties as per ASTM D 6751 standards^65^; properties being reported in Table 1. Esters formed from the trans-esterification process are the primary constituents of the biodiesel.

### Fuel blend preparation and stability

The NP concentration was fixed at 90 ppm for all nanofuel blends. This concentration was selected based on a review of existing literature, which identified it as an optimal dosage for enhancing performance and emission characteristics in similar studies, thus providing a robust basis for isolating and investigating the effect of nanoparticle size.

The BD blend composed of 20% Calophyllum Inophyllum Methyl Ester biodiesel (CIMEBD), and 80% diesel (by volume). was selected as the base blend for this study as it represents a common, commercially available blend that offers a good balance of renewable content, cost, and performance benefits, and is approved for use by numerous engine manufacturers without requiring any engine modifications.

The nanofuel blends were prepared fresh daily to ensure stability and minimize agglomeration. For each blend, the calculated mass of CeO_2_​ NPs was added to the BD base blend fuel (BD, Table 3) in a beaker. The mixture was then homogenized using a high-power ultrasonic probe sonicator (20 kHz, 400 W) for 30 min in a cooling water bath to prevent excessive heating. This process created a stable colloidal suspension that showed no visible signs of sedimentation for over 8 h, which was sufficient for the daily experimental runs. This daily preparation protocol was particularly important for mitigating the potential agglomeration of the larger 60 nm and 80 nm particles.

### Experimental details and procedure

**Fig. 2:**
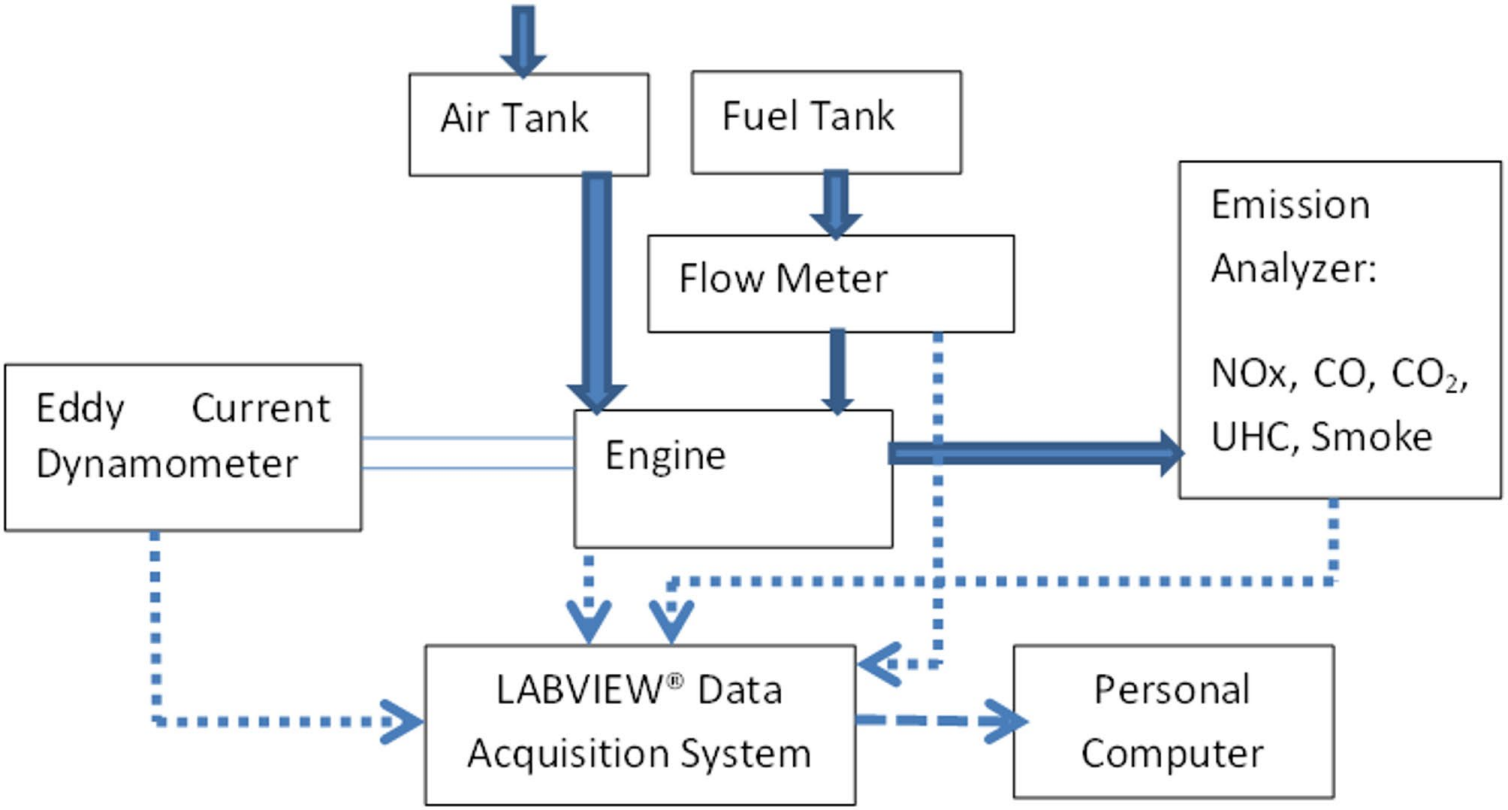
Schematic of experimental rig.

Engine details are mentioned as follows: “A naturally-aspirated, 4-stroke, single cylinder, diesel research engine equipped with an eddy-current dynamometer is used. Figure 2 shows schematic of the experimental rig, and Table 4 shows the Engine Specifications for the study. The test rig is integrated with the instrumentation for acquisition of load, temperatures, air-flow, Crank Angle (CA), and combustion pressure.” For pressure measurements, the Kistler piezoelectric pressure transducer was calibrated using a dead-weight tester prior to the experiments. During engine operation, the pressure signal for each cycle was pegged to the measured intake manifold pressure at the bottom dead center (BDC) of the intake stroke to provide an absolute pressure reference.

Table 5 lists the equipment used for measurements along with the corresponding instrument accuracy. Data is acquired by the LabView^®^ data acquisition (DAQ) system.

The engine was tested at 30% and 90% load to represent two distinct and common operating regimes. The 30% load condition simulates part-load operation, typical of urban driving, where combustion stability can be a challenge. The 90% load condition represents high-load operation, such as highway driving or acceleration, where performance is paramount. Analyzing these two points allows for a comprehensive evaluation of the fuel blends’ behavior across a wide and relevant operational range.

A fixed ratio of diesel-biodiesel blend in the ratio 20:80 is chosen, while varying the size of Ceria NP. The Ceria NP concentration in our study is fixed at 90 ppm which is the same as the optimized NP size from various earlier works as reported by Hawi et al.^66^.

For each test condition, in-cylinder pressure data were recorded for 1250 consecutive engine cycles. The raw pressure data were first ensemble-averaged to produce a mean pressure trace. This averaged trace was then processed in MATLAB using a Savitzky-Golay filtering function to remove high-frequency noise without distorting the underlying thermodynamic signal. This smoothed pressure data was then used as the input for the first-law thermodynamic model to calculate the Heat Release Rate (HRR).

**Table 3:** Fuel blends and nomenclature.

Fuel blend nomenclature	D	BD	BD20	BD40	BD60	BD80
Fuel blend specification	Diesel	20% CIMEBD, 80% Diesel	BD + NP (20 nm)	BD + NP (40 nm)	BD + NP (60 nm)	BD + NP (80 nm)

**Table 4:** Engine specifications.

Engine specifications	
Number of cylinders	1
Stroke	114.3 mm
Bore	82.5 mm
Swept volume	612 cc
Fuel system	Direct Injection
Compression ratio	(16:1)
Cooling system	Water cooled
Operating parameters	
Injection pressure	220 bar (rated)
Injection timing	23° bTDC (before Top Dead Center) (standard)
Fuel preheating	Not applied (the viscosity of the BD blend did not necessitate preheating under the tested ambient conditions)
Ambient conditions	All tests were conducted under controlled laboratory conditions (25 °C ± 2 °C, 1 atm).

Root Mean Square (RMS) error of measurements is used for estimating the measurement uncertainty from the following equation

$$\:{\mathrm{e}}_{\mathrm{R}}={\left[{\left(\frac{\partial\:\mathrm{f}}{\partial\:{\mathrm{x}}_{1}}{\mathrm{e}}_{1}\right)}^{2}+{\left(\frac{\partial\:\mathrm{f}}{\partial\:{\mathrm{x}}_{2}}{\mathrm{e}}_{2}\right)}^{2}+\cdots\:+{\left(\frac{\partial\:\mathrm{f}}{\partial\:{\mathrm{x}}_{\mathrm{n}}}{\mathrm{e}}_{\mathrm{n}}\right)}^{2}\right]}^{\frac{1}{2}}$$.

, where e_R_ is the uncertainty in result R, and x_i_ are measured variables.

### Statistical analysis

To ensure the statistical validity of the experimental results, all tests for each operating condition were repeated three times, and the data are presented as the mean ± standard deviation (SD). To determine whether the differences observed among the six fuel groups (Diesel, BD, BD20, BD40, BD60, and BD80) were statistically significant, a one-way Analysis of Variance (ANOVA) was performed. A p-value of less than 0.05 was considered statistically significant. Following a significant ANOVA result, Tukey’s Honestly Significant Difference (HSD) post-hoc test was conducted to perform pairwise comparisons and identify which specific fuel groups differed significantly from one another. The results of these tests are reported in the relevant sections of the results and discussion.

**Table 5:** Instrument range/accuracy.

Instrument	Range/accuracy/uncertainty
Coriolis flow meter	0–240 kg/h ; ±0.1% of measured value
Load cell dynamometer	0-1200 Nm; ±0.25% of full scale
K-type thermocouple	± 0.75%;-200–1250 deg. C

## Results and discussion

### Combustion stability (COVIMEP)

COVIMEP is an important parameter used for characterizing the cycle-to-cycle fluctuations of the combustion process, and is a measure of combustion stability. The COVIMEP should not be more than 5–10% for stable operation^67,68^.

It is calculated as:

$$\:{\mathrm{C}\mathrm{O}\mathrm{V}}_{\mathrm{x}}=\frac{\sqrt{\left({\sum\:}_{\mathrm{i}=1}^{\mathrm{N}}{({\mathrm{x}}_{\mathrm{i}}-\stackrel{-}{\mathrm{x}})}^{2}/\mathrm{N}\right)}}{\stackrel{-}{\mathrm{x}}}\cdot 100\%,\:\:\:\:\mathrm{w}\mathrm{h}\mathrm{e}\mathrm{r}\mathrm{e}\:\stackrel{-}{\mathrm{x}}=\frac{1}{\mathrm{N}}\sum\:_{\mathrm{i}=1}^{\mathrm{N}}{\mathrm{x}}_{\mathrm{i}}\:$$

**Fig. 3:**
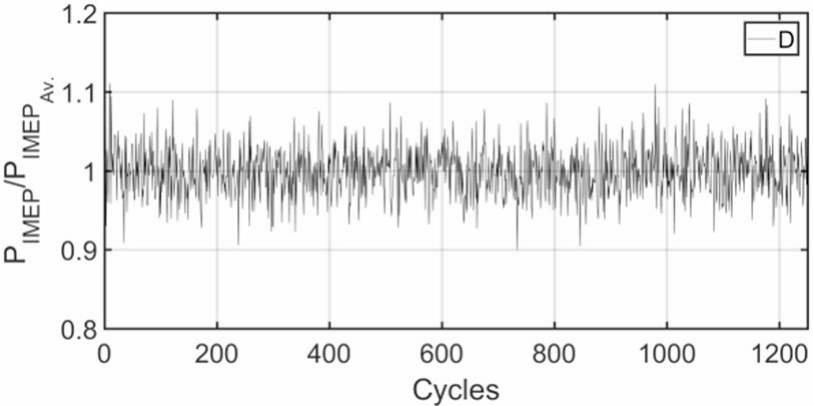
P_IMEP_ variation for D (Diesel); 30% load.

**Fig. 4:**
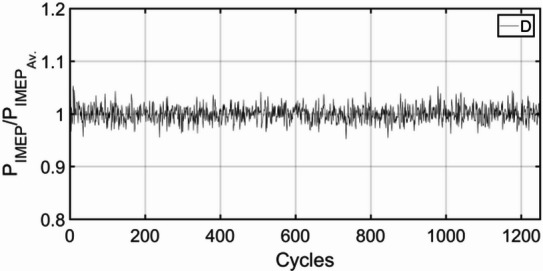
P_IMEP_ variation for D (Diesel); 90% Load.

**Fig. 5:**
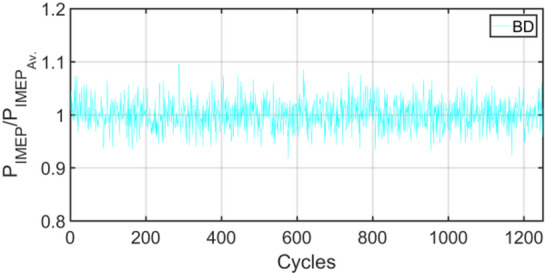
P_IMEP_ variation for BD; 30% Load.

**Fig. 6:**
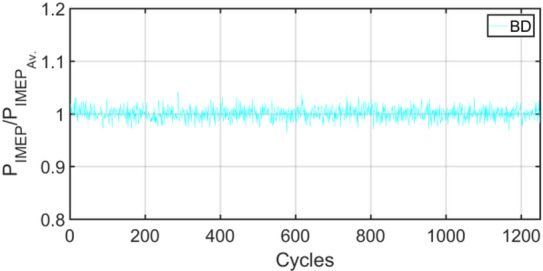
P_IMEP_ variation for BD; 90% Load.

**Fig. 7:**
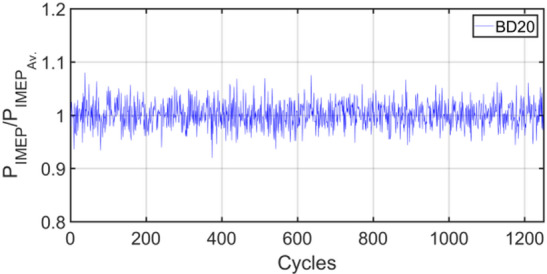
P_IMEP_ variation for BD20; 30% Load.

**Fig. 8:**
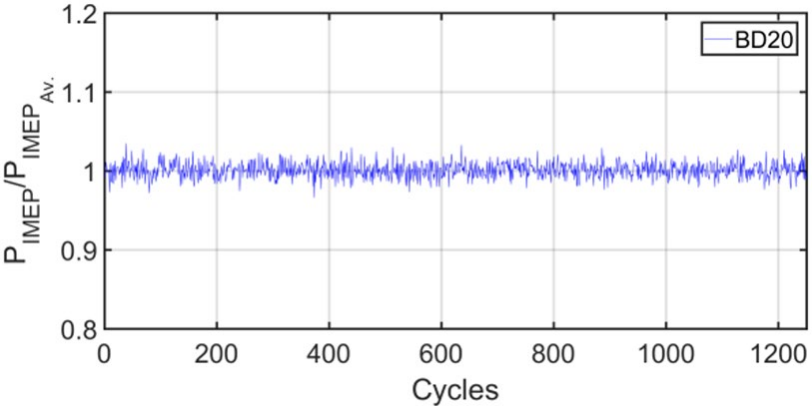
P_IMEP_ variation for BD20; 90% Load.

**Fig. 9:**
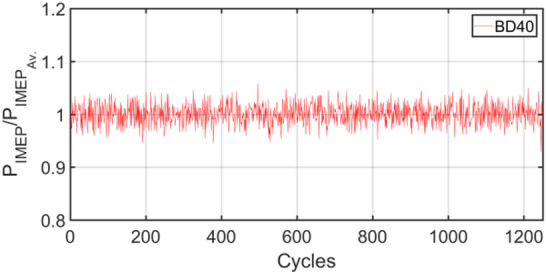
P_IMEP_ variation for BD40; 30% Load.

**Fig. 10:**
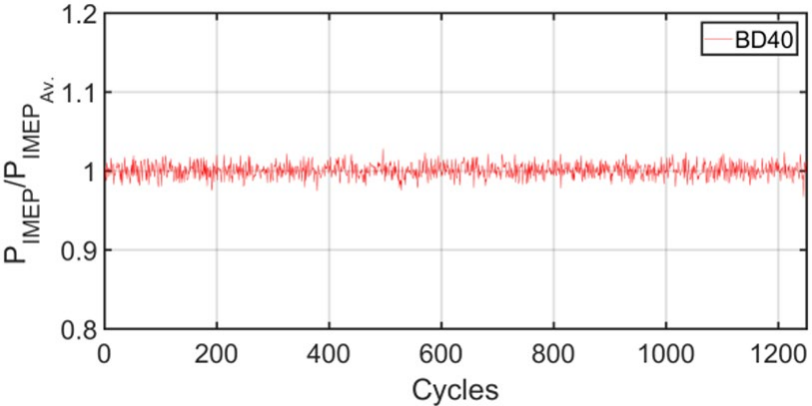
P_IMEP_ variation for BD40; 90% Load.

**Fig. 11:**
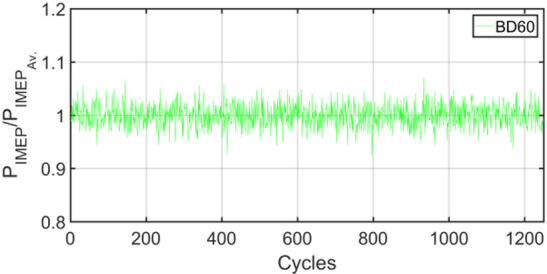
P_IMEP_ variation for BD60; 30% Load.

**Fig. 12:**
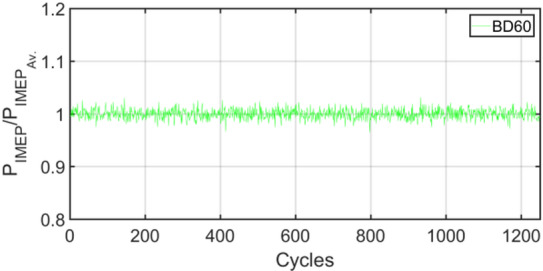
P_IMEP_ variation for BD60; 90% Load.

**Fig. 13:**
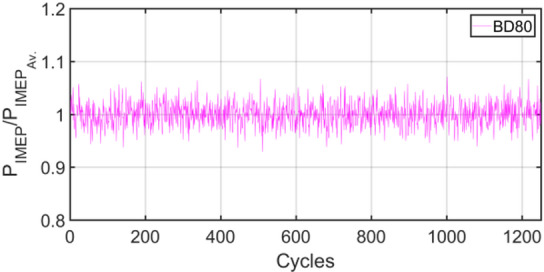
P_IMEP_ variation for BD80; 30% Load.

**Fig. 14:**
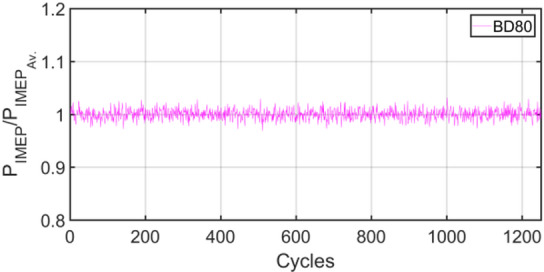
P_IMEP_ variation for BD80; 90% Load.

**Fig. 15:**
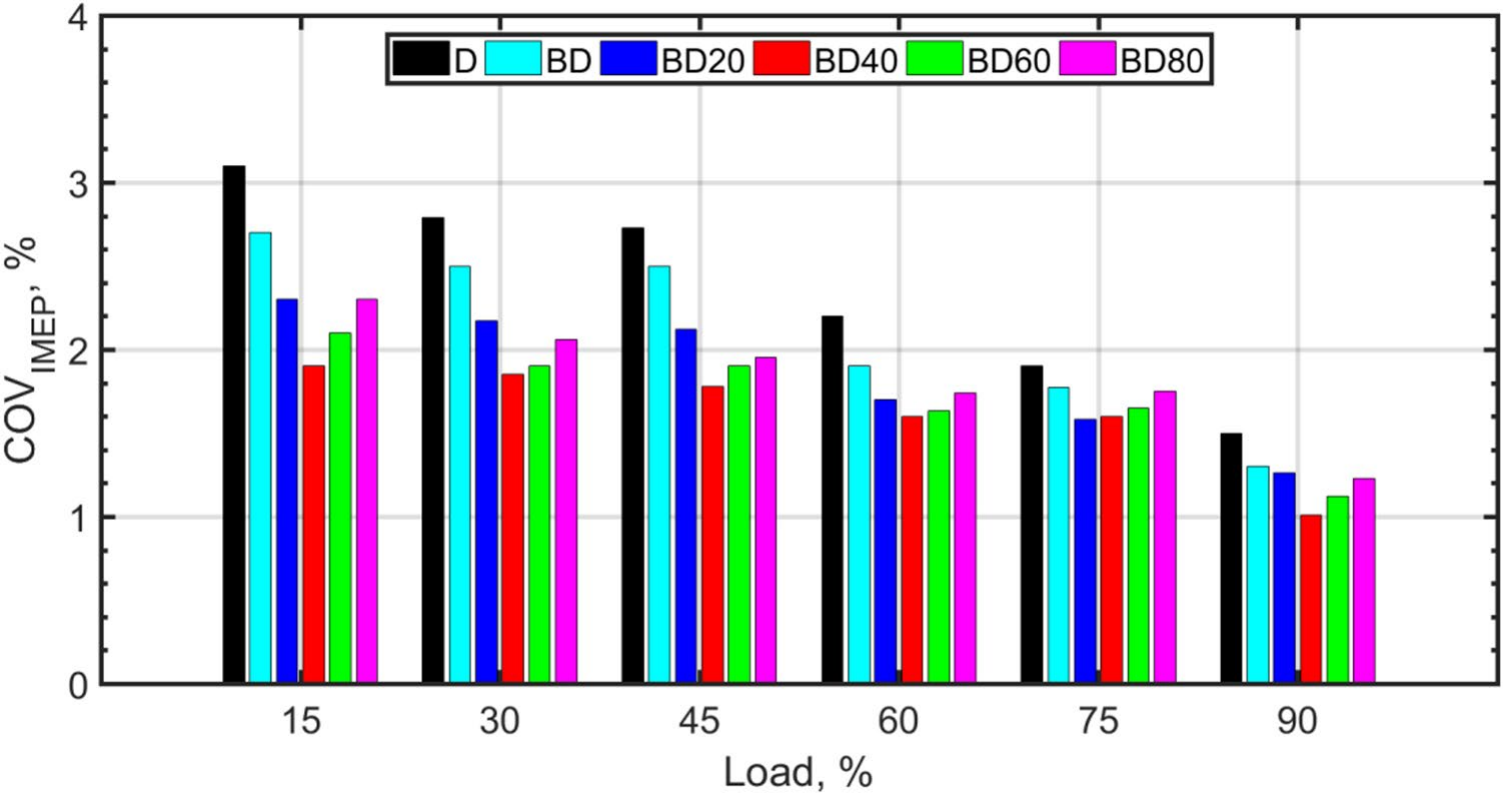
COVIMEP variation for the fuel blends under various loads.

Figures 3, 4, 5, 6, 7, 8, 9, 10, 11, 12, 13 and 14 shows cyclic variations of peak pressure normalized by average value of peak pressure over 1250 cycles. The figure presents the cyclic variations of the indicated mean effective pressure (PIMEP​), normalized by the average value over 1250 cycles, for all fuel blends at 30% and 90% load. This plot format visualizes the statistical distribution of combustion stability. The COVIMEP has been calculated from the data presented in the Figs. 3, 4, 5, 6, 7, 8, 9, 10, 11, 12, 13 and 14 plots. Figure 15 shows COVIMEP for the various tested fuels and for two load conditions, namely 30% load (low load), and 90% load (high load). The values are presented and demonstrated for 1250 complete engine working cycles. At 90% load, the mean COVIMEP​ for the BD40 blend was 1.9% ± 0.2%, compared to 3.1% ± 0.3% for conventional diesel. A similar trend was observed at 30% load, where the BD40 blend had a mean COVIMEP of 2.5% ± 0.3% versus 3.8% ± 0.4% for diesel. An ANOVA confirmed that the differences in COVIMEP​ among the fuel groups were statistically significant (*p* < 0.001) at both load conditions. Tukey’s post-hoc analysis revealed that at 90% load, the COVIMEP​ for the BD40 blend was significantly lower than that for both conventional diesel and the baseline B20 blend (*p* < 0.05). It is observed for the low load condition that the COVIMEP is highest for pure diesel, whereas it decreases for BD, BD20, BD40, BD60, and BD80, in that order. COVMEP and ID values are given in Table 6. COVIMEP is shown to be in direct correlation with ID, which is the time between start of injection (SOI) to start of combustion or ignition. The short period immediately following this ID is a premixed combustion phase and this is followed by a mixing-controlled, diffusion combustion phase^69^. The mixing-controlled combustion has little effect on the cyclic variability, whereas the short, premixed combustion phase has a predominant effect on the cyclic variability. Furthermore, the ID has direct correlation with COVIMEP. A short ID leads to smaller COVIMEP^70^. This has been corroborated by Yang et al.^71^ also. Furthermore, the higher CN of Biodiesels causes a decrease in ID, and therefore, a decrease in COVIMEP. It has been also shown by Icingur et al.^28^ that higher CN causes a decrease in ID in diesel engines. The results in Fig. 15, and Table 6 indicate that the COVIMEP is lowest for BD40. Also, the COVIMEP decreases with load. At higher load, due to larger in-cylinder peak pressures, and corresponding higher temperatures, the ID is reduced. This can be confirmed from the results of Yang et al.^71^ who have done simulations for ID for various test fuels, and found that ID decreases with increasing temperature. Due to larger CN of BD40, it has smaller COVIMEP compared to diesel. Also, for higher loads the COVIMEP reduces for all the fuels of concern and the differences are the same as that for low loading, although less significant. This trend was confirmed by Yang et al.^71^ also. Yasin et al.^72^ has also shown that COVIMEP decreases with increase in peak pressure and also with increasing load. Jeyaseelan et al.^73^ had investigated biodiesel from coconut and found that COVIMEP decreases with increasing load and increases with increasing rpm. However, the results for biodiesel and diesel were not clearly demarcated. Gorski et al.^74^ reported high COVIMEP with more di-ethyl-ether (DEE) fraction. For the case of NP, the cyclic variability decreases with increase in NP size, up to 40 nm size. The results show that COVIMEP are lower for the NP blended fuels. With the increase of NP concentration, it is seen that COVIMEP reduces. However, it is the lowest for 40 nm particles and thereafter the change is not significant. Therefore, the BD with 40 nm particles is most suitable for decreasing cyclic variability. The reason may be attributed to the fact that larger sized NPs have agglomeration issues and may not be as effective in aiding the ignition process compared to smaller sized NPs^62^. So COVIMEP decreases with load, increases with biodiesel content and also decreases with NP size up to between 20 and 40 nm, and increased thereafter.

The importance of the study of cyclic variability is heightened by the fact that various combustion strategies are being investigated, such as LTC, HCCI, PCCI, and RCCI. These techniques involve sufficient charge dilution, which limits their cyclic variability^75^. To allow a proper utilization of such modes along with the use of newer fuel types such as nanofuels used in this study, a proper understanding of the cyclic variability is needed.

**Table 6:** COVIMEP and ID at 30%, and 90% load.

	D	BD	BD20	BD40	BD60	BD80
COVIMEP, %						
30% Load	3.1	2.7	2.3	1.9	2.1	2.3
90% Load	1.5	1.1	0.86	0.75	0.77	0.79
ID (crank angle degrees)						
30% Load	4.5	3.6	3.23	3.14	3.28	3.33
90% Load	3.37	2.7	2.48	2.25	2.4	2.55

### Ignition characteristics (ID and CN)

**Fig. 16:**
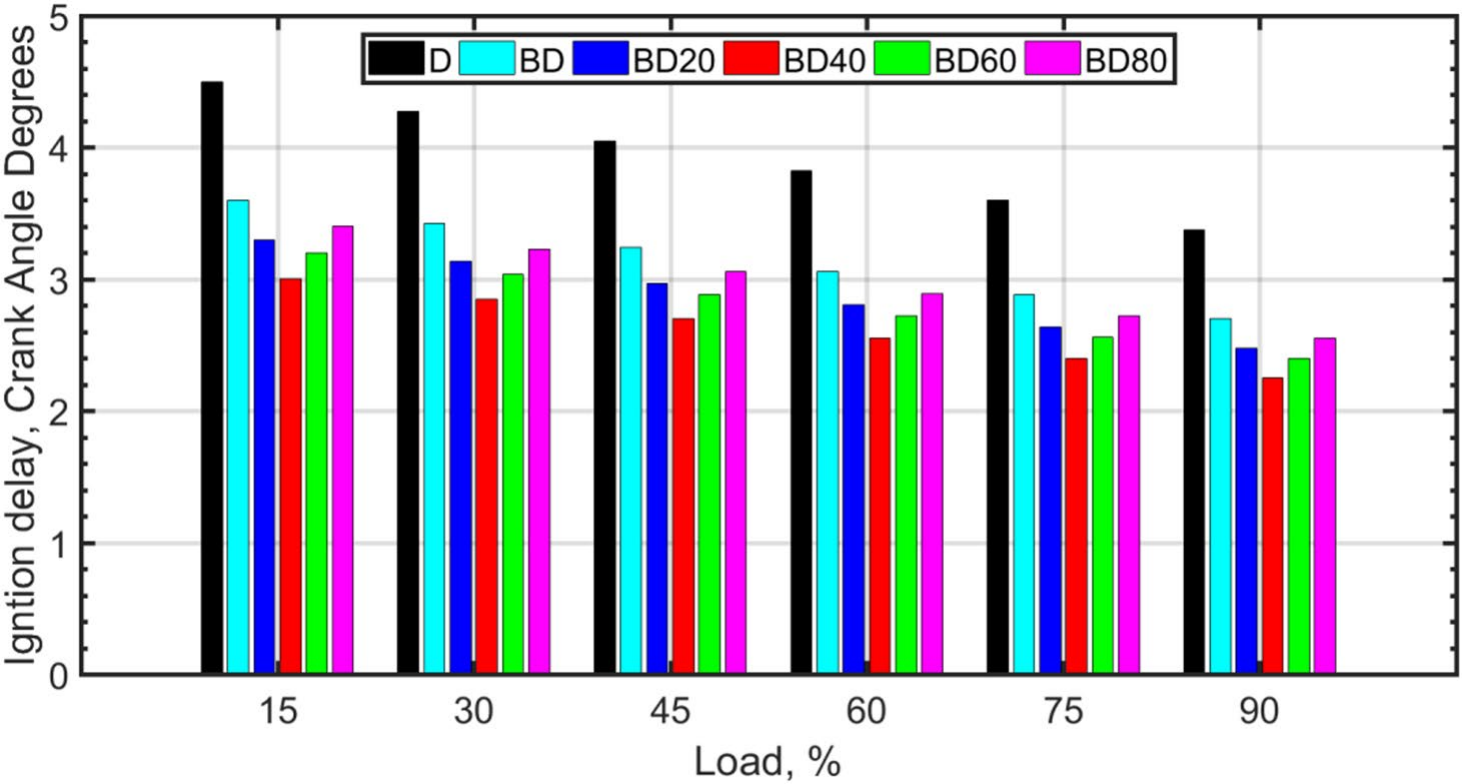
Variation of ID (CAD) with fuel type and load.

ID is defined as the period between start of injection (SOI) to the start of combustion (SOC). In terms of CAD, it is the point where a positive slope of HRR is observed. The importance of ID is emphasized in various investigations. The results of Fig. 16 tabulated for a range of engine loads, show that the ID decreases with load. The decrease of ID for biodiesel (BD) and for nanofuels BD20 to BD80 is also evident. Biodiesels have higher CN and the presence of molecular oxygen, which are known to lower the ID^28,71^.

The catalytic effect of the nanoparticles led to a marked reduction in ignition delay (ID) across all conditions. At 30% load, the BD40 blend exhibited a mean ID of 2.7 ± 0.3 CAD, significantly shorter than the 4.5 ± 0.4 CAD for conventional diesel. This effect persisted at high load, where the ID for BD40 was 2.1 ± 0.2 CAD compared to 3.5 ± 0.3 CAD for diesel. The effect of fuel type on ignition delay was found to be statistically significant (*p* < 0.001) via ANOVA. Specifically, Tukey’s HSD test showed that the ignition delay for the BD40 blend was significantly shorter than for all other fuel blends and conventional diesel at both 30% and 90% loads (*p* < 0.05).

It can be observed that for the low loads, the change in ID is substantial, whereas for the higher loads, the change is not so significant. The change in ID of various fuels with respect to diesel shows that the ID is not so affected at high loads. The reason may be attributed to the higher pressures and corresponding temperatures at high loads. The higher temperatures induce quicker evaporation, fuel-air mixing, and faster reaction kinetics, and thereby contribute towards the reduction in ID. Therefore, the effect of CN towards the reduction in ID is not so evident at higher temperatures. It has also been shown by Yang et al.^71^ that for high temperatures corresponding to high loads (or pressures), the ID, in milliseconds, is known to decrease. For the same RPM, this can be interpreted as a decrease in ID (in CADs). The effect of NP additives on the ID is also shown in Fig. 16. NP additives reduce the ID further, as they have catalytic sites on their surface that assist in chemical reaction kinetics that occur during the pre-ignition phase, and also they are known for ‘microexplosion’ phenomenon that assists local fuel-air mixing.

The effect of CN on ID is also a subject of investigation. The CN are evaluated from the standard procedure as follows. The standard ASTM D613 is used for determining CN. The reference fuels for calculating the CN are n-cetane (n-C_16_H_34_), and 2,2,4,4,6,8,8-heptamethylnonane, with CNs 100, and 15, respectively.

The calculated CN for the BD40 blend was 55.4 ± 0.5, which was higher than that of conventional diesel (49.1 ± 0.4). Statistical analysis confirmed that the differences in the calculated CNs were significant (*p* < 0.001). The CN of the BD40 blend was found to be significantly higher than that of all other fuels tested (*p* < 0.05), which directly corresponds to its shorter ignition delay.

For a fuel that has the same ID period as a mixture of the two reference fuels, the CN is calculated from the formula, CN = % n-cetane + 0.15 (% heptamethylnonane). The CNs have been measured as 49.1, 51.6, 52.5, 55.4, 54.1, and 53.7 for D, BD, BD20, BD40, BD60, and BD80, respectively. CNs are the highest for BD40, and then decrease slightly for BD60 and BD80. The correlation of CN with COVIMEP, and ID can be seen to match with the trend of CN. Icingur et al.^28^ showed that CN increase leads to a decrease in ID. The CN with higher NP size is slightly lower, and this may be attributed to the agglomeration of larger sized particles and the difficulty of keeping large NP colloidal mixtures stable for a longer period of time.

Various investigations have revealed that the ID is known to decrease with CN. Kyrtatos et al.^70^ observed that CN is an important parameter affecting the ID. They verified that it is the premixed combustion duration, i.e., the combustion immediately after the ID that is responsible for cycle-to-cycle variations of the engine. The premixed combustion duration is followed by the mixing-rate controlled diffusion combustion, which has little to do with causing the cyclic variability in the engine. Even though for CI engines, the cyclic variations are small, they are to be investigated when newer combustion strategies are being researched. The premixed combustion duration is usually small (about 25% of the entire combustion duration). However, it may be larger under some circumstances^76^. Icingur et al.^28^ observed a direct correlation between CN and ID. Kyrtatos et al.^70^ have shown that for fuels with larger CN, the ID is lower, and this also results in lower cyclic variability. These results have also been confirmed by Pham et al.^77^, who further noted that the ID is composed of a physical, and a chemical duration. Apart from the ID duration, the variability in ID also plays a role in the engine cyclic variability^70,78^.

### In-cylinder pressure and heat release rate

**Fig. 17:**
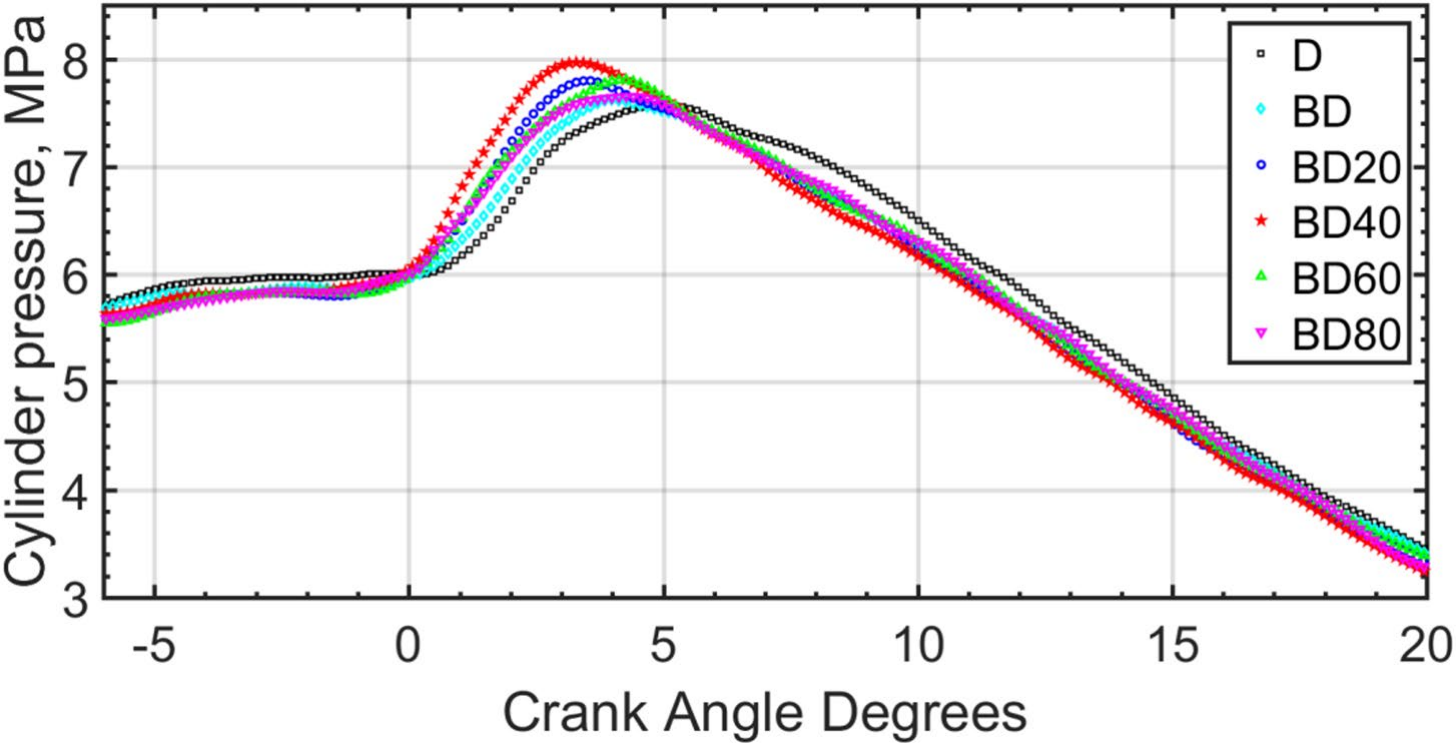
In-cylinder pressure rise for the various fuel blends; 1500 RPM, part load.

The pressure within the cylinder is measured using a pressure transducer, and the HRR is calculated from it by thermodynamic model^69^. The pressure curves for the six fuel blends are shown for 1500 RPM, and part load operation in Fig. 17. The x-axis represents the engine Crank Angle in Degrees (°), where 0° corresponds to Top Dead Center (TDC). For each test condition, in-cylinder pressure data were recorded for 1250 consecutive engine cycles. The raw pressure data were first ensemble-averaged to produce a mean pressure trace. This averaged trace was then processed in MATLAB using a Savitzky-Golay filtering function to remove high-frequency noise without distorting the underlying thermodynamic signal. The pressure curves indicate that the maximum pressure is attained for BD40, and also it is closest to TDC. The early pressure rise for biodiesels compared to diesel, especially for BD40 shows that early ignition corresponding to a short ID and corresponding large CN is indicated from the figure. However, for the case of BD60, and BD80 the pressure rise is slightly retarded compared to BD40. The reason may be due to the lower CN, with higher NP concentration, which further stems from the fact of NP agglomeration in the fuel and a resulting unstable fuel suspension for NPs. This is one of the disadvantages of the CeO_2_ NPs and needs further research.

**Fig. 18:**
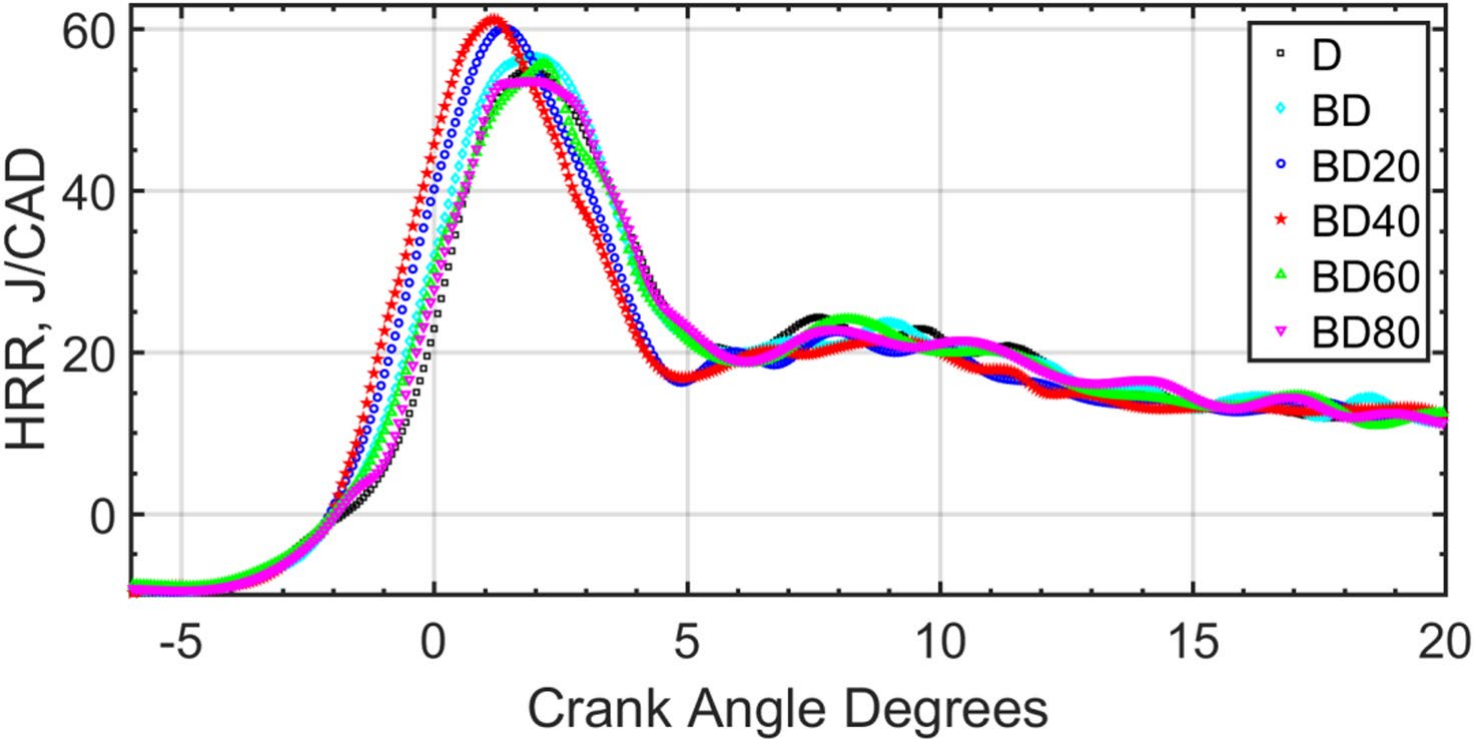
HRR for fuel blend at 1500 RPM, part load.

Figure 18 shows the HRR for the fuel blends. As mentioned earlier, the HRR is derived from the pressure curve by thermodynamic calculations. It is seen from the curves that BD40 has the earliest pressure rise corresponding to higher CN, and corresponding lower ID, as detailed in the earlier discussion for ID, and CN. Similar logic as to COVIMEP applies to NP size effect and the observed performance. The most desirable performance is shown for BD40, i.e., BD blended with 40 nm NPs.

**Fig. 19:**
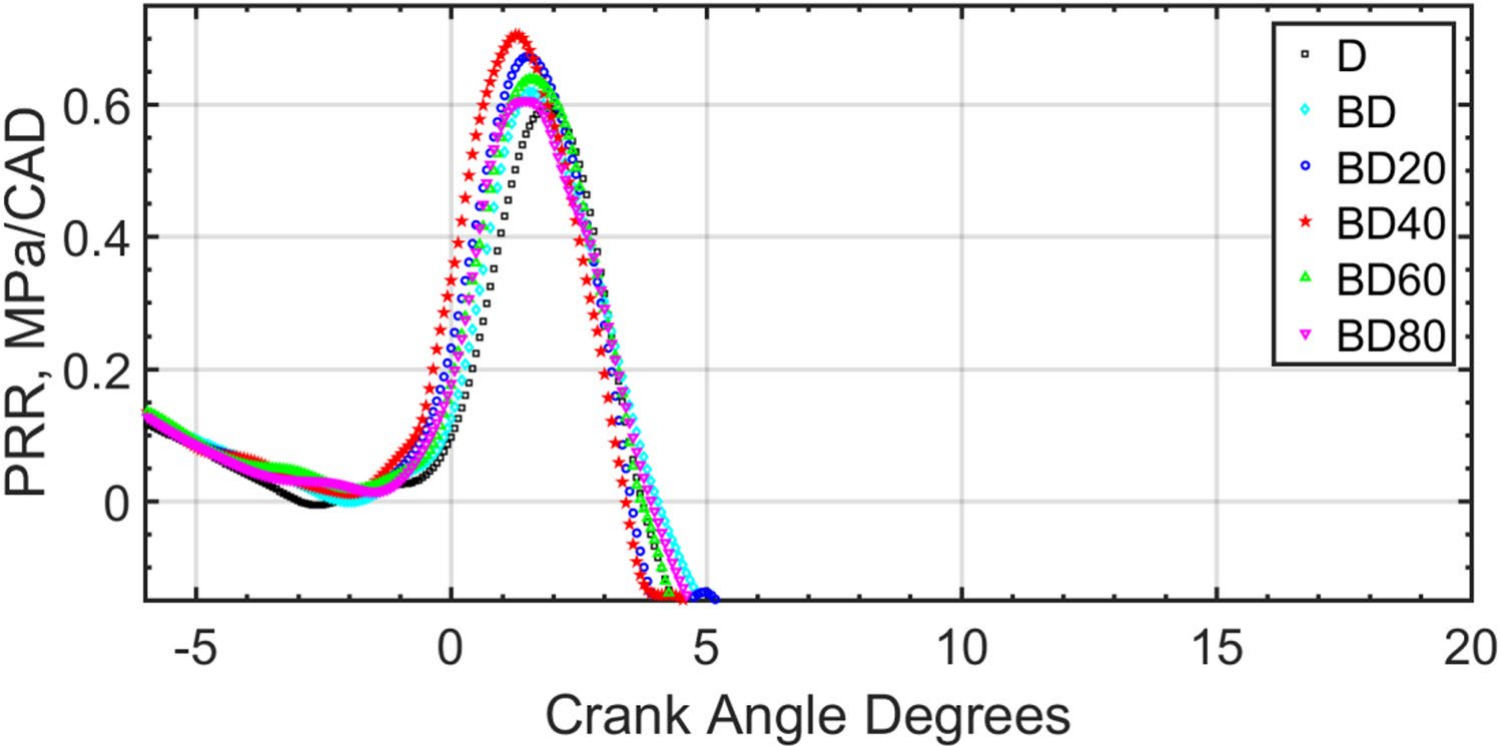
Pressure rise rate for the fuel blends for 1500 RPM, part load.

The max cylinder pressure increases with load and is confirmed with results of Jeyaseelan et al.^73^. Representative pressure and HRR curves are shown for part load operation in the figures. The pressure rise rate is shown in Fig. 19. The pressure rise rate can easily be calculated from the discrete values of pressure and CAD. Similar to the earlier pressure, and HRR rates, this figure also shows higher pressure rise rate for BD40 compared to all the other test fuels.

An ANOVA confirmed that the differences in peak pressure were statistically significant (*p* < 0.05). The peak in-cylinder pressure for the BD40 blend was significantly higher than that for both diesel and the baseline BD blend, as determined by a Tukey’s post-hoc test. The optimality of the 40 nm particle size is attributed to a balance between catalytic surface area and particle stability. As size increases from 20 nm to 40 nm, the catalytic effect is enhanced. However, beyond 40 nm, van der Waals forces lead to greater particle agglomeration. These larger clusters reduce the effective surface-area-to-volume ratio, diminishing their catalytic impact and hindering uniform dispersion, as reflected in the slightly retarded combustion phasing for BD60 and BD80.

## Conclusions

An investigation is conducted with the fuel blend Calophyllum Inophyllum Methyl Ester biodiesel (20%), and diesel (80%) along with CeO_2_ NP additives of sizes 20 nm, 40 nm, 60 nm, and 80 nm. A single cylinder, 4-stroke CI engine was used for the purpose. The size effects of the NP on combustion characteristics such as COVIMEP, ID, CN, In-cylinder pressure rise, HRR, and pressure rise rate were investigated at various load conditions, and a constant engine RPM of 1500.

The COVIMEP, which is an indicator of engine cyclic variability or alternately, combustion stability, decreases at higher engine load. For higher biodiesel fraction fuel blend, i.e. for BD, compared to pure Diesel, the COVIMEP was lower. COVIMEP further decreased with NP size and the optimum COVIMEP was found for BD40 (20% biodiesel-80% diesel, 40 nm CeO_2_, at 90 ppm). The differences in COVIMEP were more predominant for low loads, compared to the higher loads.

Another observation is that ID is in inverse correlation to the CN. Increase in ID showed a larger engine cyclic variability, whereas the opposite was observed with lower ID fuels. Biodiesel addition to diesel caused an increase in CN, and a decrease in ID. NPs further increased the CN and lowered the ID. The highest CN fuel was BD40 (20% biodiesel-80% diesel-40 nm CeO_2_, at 90 ppm), which also had the smallest ID. COVIMEP is also related inversely to ID. Fuel blends with higher ID showed a lower COVIMEP. The ID plays an important role in determining the cyclic variability of the engine, and is even more important for lean operating conditions. The effect of various diluents, and additives needs to be investigated further in relation to the effect on combustion characteristics, and engine cyclic variability, which is an important parameter for engine stability.

In summary, the principal finding of this study is that nanoparticle size has a significant and non-linear effect on the combustion stability of CIMEBD blends. An optimal size of 40 nm for CeO_2​_ NPs was found to provide the greatest reduction in cyclic variability by minimizing ID, a result that is strongly correlated with the measured increase in the fuel CN.

### Practical implications

The findings of this study offer several practical implications. The demonstrated improvement in combustion stability (i.e., lower COVIMEP​) with the BD40 blend translates directly to smoother engine operation, which can reduce noise, vibration, and harshness (NVH), leading to improved driver comfort and potentially longer engine component life. For engine designers, greater combustion stability opens the door for implementing more advanced and efficient combustion strategies, such as Low-Temperature Combustion (LTC), which are often limited by instability. Furthermore, our identification of an optimal nanoparticle size (40 nm) provides crucial guidance for fuel additive manufacturers. It suggests that simply increasing nanoparticle size does not guarantee better performance and can, in fact, be counterproductive due to agglomeration, thus preventing the inefficient and costly use of larger nanoparticles. For fleet operators and other end-users, the adoption of an optimized nano-fuel blend like BD40 could lead to tangible benefits in fuel economy and reduced maintenance related to unstable combustion.

### Environmental and health considerations of nanoparticle emissions

While this study demonstrates the combustion benefits of CeO_2_​ NP additives, it is important to address their responsible implementation. The combustion process does not destroy the nanoparticles; rather, they are emitted in the exhaust, often with altered physical and chemical properties. The potential environmental and health effects of such emissions are a known consideration in the field. Therefore, an effective path forward for leveraging the advantages of fuel-borne catalysts involves their use in conjunction with advanced exhaust after-treatment systems. Future research could also explore composite nanoparticles, such as combining CeO_2_ with materials reported to have a lower environmental impact, like carbon nanotubes (CNTs), to potentially mitigate risks while retaining catalytic benefits. Technologies such as Diesel Particulate Filters (DPFs) are designed to capture particulate matter, and their effectiveness in trapping metallic NPs is a critical area of study. The integration and optimization of such after-treatment methods to specifically manage NP emissions would ensure that the performance and efficiency gains are realized while mitigating potential risks. This approach, which is already a regulatory requirement in some regions, represents a vital focus for future research to enable the widespread and safe application of nano-fuel technology.

### Recommendations for future studies

Based on the findings of this work, the following areas are recommended for future investigation:

1. Optimization of NP Concentration: Investigate the effect of varying the concentration of the optimal 40 nm CeO_2_ NPs to determine the ideal dosage for maximizing combustion stability.
2. Long-Term Durability Studies: Conduct long-duration engine tests to assess the long-term impacts of these nanofuels on engine wear, injector fouling, and lubricating oil degradation.
3. Investigation of After-Treatment Systems: As discussed, the use of fuel-borne catalysts should be paired with effective after-treatment. Future work should study the efficiency of modern Diesel Particulate Filters (DPFs) in capturing the emitted metallic nanoparticles.
4. Broader Applicability: Extend this research to other types of biodiesel and different NP materials (e.g., Al_2_O_3_, CNTs) to determine if the size-dependent effect on combustion stability is a general phenomenon.

## Data Availability

The datasets used and/or analysed during the current study available from the corresponding author on reasonable request.

## Abbreviations

ASTM: American society for testing and materials
BD: Biodiesel-diesel blend (20% CIMEBD, 80% Diesel)
BD20, BD40, BD60, BD80: BD blend with 20, 40, 60, 80 nm NPs
BSFC: Brake specific fuel consumption (kg/kWh)
BTE: Brake thermal efficiency (%)
CA / CAD: Crank angle/crank angle degrees (°)
CeO2​: Cerium oxide
CI: Compression ignition
CIMEBD: Calophyllum inophyllum methyl ester biodiesel
CN: Cetane number
CO: Carbon monoxide
CO2​: Carbon dioxide
COVIMEP​: Coefficient of variation of indicated mean effective pressure (%)
D: Diesel
DAQ: Data acquisition
FFA: Free fatty acid
HC / UHC: Hydrocarbons/unburnt hydrocarbons
HCCI: Homogeneous charge compression ignition
HRR: Heat release rate (J/CAD)
ICE: Internal combustion engine
ID: Ignition delay (CAD)
LTC: Low-temperature combustion
NP: Nanoparticle
NOx: Oxides of nitrogen
PCCI: Premixed charge compression ignition
PRR: Pressure rise rate (MPa/CAD)
RCCI: Reactivity controlled compression ignition
RPM: Revolutions per minute
SOC: Start of combustion
SOI: Start of injection
TDC: Top dead center
XRD: X-Ray diffraction
